# Patient experiences of hospital care during the COVID-19 pandemic in Ireland: Daniela Rohde

**DOI:** 10.1093/eurpub/ckac131.282

**Published:** 2022-10-25

**Authors:** D Rohde, C Foley, R Murphy, M Kelly, L Drummond

**Affiliations:** National Care Experience Programme, Health Information and Quality Authority, Dublin, Ireland

## Abstract

**Introduction:**

The COVID-19 pandemic has greatly impacted healthcare service delivery. This study explored patient experiences of hospital care during the COVID-19 pandemic in Ireland, using National Inpatient Experience Survey (NIES) 2021 data.

**Methods:**

NIES is a repeat cross-sectional survey of inpatient experiences in all public acute hospitals in Ireland. Patients who spent 24+ hours in hospital and were discharged in September 2021 were eligible to participate. 7 questions addressed experiences specific to the pandemic. Comparisons between 2019 and 2021 were conducted using t-tests. Effect sizes (d) are reported. Qualitative data were thematically analysed.

**Results:**

10,743 patients participated (42% response rate). While 68% did not feel at risk of catching COVID-19, 9% felt at risk. 35% reported that staff always helped them to keep in touch with family. There were small, statistically significant differences between 2019 and 2021 ratings, with questions on opportunity for family to talk to a doctor (d=-.328), provision of information to family (d=-.136), and being able to find staff to talk to about worries and fears (d=-.167) recording the biggest decreases. Scores for cleanliness of wards (d = 0.063) and bathrooms (d=.075), and privacy during examination or treatment in the ED (d = 0.085) improved significantly. Patients commented on their appreciation of staff, but missed having visitors, with restrictions posing challenges for those with sensory or physical impairments.

**Conclusions:**

Given the unique challenges experienced by acute healthcare services during the COVID-19 pandemic, comparisons with pre-pandemic patient experiences should be interpreted with caution. Continuing to gather patient feedback during a pandemic presents a unique opportunity to understand the resilience of healthcare systems as they continue to operate under unprecedented pressure, with the potential to inform responses and delivery of care during future pandemics or other emergencies.

**Key messages:**

• Visiting restrictions posed many challenges for patients and affected communication both between patients and their family members, as well as between healthcare staff and patients’ family.

• Gathering patient feedback during a pandemic presents a unique opportunity to inform responses and delivery of care during future pandemics or other emergencies.

